# Effect of the Binder during Ultra-Precision Polishing of Tungsten Carbide Using a Semirigid Bonnet Tool

**DOI:** 10.3390/ma15238327

**Published:** 2022-11-23

**Authors:** Xiaolong Ke, Wei Wu, Kangsen Li, Yongheng Yu, Tianyi Wang, Bo Zhong, Zhenzhong Wang, Jiang Guo, Chunjin Wang

**Affiliations:** 1 School of Mechanical and Automotive Engineering, Xiamen University of Technology, Xiamen 361024, China; 2 State Key Laboratory of Ultra-Precision Machining Technology, Department of Industrial and Systems Engineering, The Hong Kong Polytechnic University, Hung Hom, Kowloon, Hong Kong, China; 3 National Synchrotron Light Source II (NSLS-II), Brookhaven National Laboratory, P.O. Box 5000, Upton, NY 11973, USA; 4 Department of Automation, Tsinghua University, Beijing 100084, China; 5 School of Aeronautics and Astronautics, Xiamen University, Xiamen 361005, China; 6 Key Laboratory for Precision and Non-Traditional Machining Technology of Ministry of Education, Dalian University of Technology, Dalian 116024, China

**Keywords:** tungsten carbide, bonnet polishing, surface integrity, material removal, binder, ultra-precision machining

## Abstract

Tungsten carbide (WC) has the characteristics of high hardness, high strength, corrosion resistance, wear resistance and excellent fracture toughness. Accordingly, it has been commonly used as the material for cutting tools and molds in glass-forming techniques. To obtain ultra-smooth surfaces, fine polishing of WC is indispensable. However, the efficiency of WC polishing is low using the existing polishing methods, and the mechanism behind the polishing process requires further investigation. Specifically, the effect of the binder in WC polishing is not clear since there are different kinds of WC with various weight percentages of the binder. In this paper, we present the findings of a study on the polishing performance of two kinds of WC material, with and without the binder, using a semi-rigid (SR) bonnet polishing tool. A series of experiments were performed on a 6-DOF robotic polishing instrument to investigate the material-removal characteristics, surface integrity and sub-surface damage after polishing. The results demonstrate that the SR bonnet polishing tool successfully reduced the surface roughness of WC with and without the binder to the nanometric level, though the lowest surface roughness was obtained on binder-less WC. No obvious sub-surface damage was observed under SEM inspection, while the processing efficiency was greatly improved owing to the high material removal rate of the tool. Based on our analysis of key polishing parameters and corresponding surface integrities, the effect of the binder on the polishing performance is explained, which offers excellent guidance for WC polishing.

## 1. Introduction

Tungsten carbide (WC) is one of the most important materials used for scientific research, industrial molds and products, and precision tools [1,2,3], owing to its high hardness, high strength, corrosion resistance [4,5,6], wide range of wear resistance and good fracture toughness [7,8,9]. Among the different kinds of WC, binder-less tungsten carbide (termed ‘WC-0Co’) is mainly used for the manufacture of precision molds in the fields of optics and aerospace [10]. As a common binder of cemented carbide, cobalt (Co) has good adhesion to WC [11,12]. The WC phase gives the alloy excellent hardness and wear resistance, while the binder phase determines the strength and toughness of the composite [13]. Liu et al. [14] manufactured a tungsten carbide alloy with 4 wt.%~14 wt.% Co content via spark plasma sintering and explored the effect of binder Co on the properties of tungsten carbide; the results showed that, as the Co content increased, the density, fracture toughness and flexural strength of cemented carbide increased, but the hardness gradually decreased. In addition, Hosseini et al. [15] investigated the effect of Co addition on the microstructural and mechanical properties of WC-B4C-SiC composites sintered using the spark plasma sintering (SPS) method, and indicated a sample containing 10 vol.% Co obtained excellent flexural strength, fracture toughness and hardness. WC-Co cemented carbide has become commonly used given its excellent combination of mechanical properties with hardness and toughness, and it is now the main material for die manufacturing [16,17]. In the industry, there is a vast demand for WC as it is used in molds for high-precision glass lenses. To improve the surface quality of the molds and meet the ever-increasing precision requirements, an effective and efficient polishing method of WC molds is thus indispensable [18].

At present, the common mold-polishing methods include bonnet polishing, fluid jet polishing (FJP), magnetorheological polishing, etc. Beaucamp et al. [19] studied the surface integrity of WC-0Co using FJP, and the results revealed that a large abrasive size and low fluid pressure were useful for the polishing of WC-0Co to reduce the surface roughness and abrasive embedment. Deng et al. [20] implemented ultra-smooth polishing of WC through the combination of anodizing and soft abrasive polishing, in which anodizing helped soften the WC workpiece to increase the material removal rate. Wang et al. [21] proposed a magnetorheological polishing instrument for optical molds, aiming at suppressing the mid-spatial frequency errors caused by ultra-precision grinding. Jahan et al. [22] carried out the fine-finishing of WC using die-sinking micro-electro-discharge machining. However, it was difficult to apply these machining processes without creating surface and sub-surface (i.e., thermal) damage. Kim and Lee [23] performed finishing of sintered carbide through grinding with an in-process electrolytic dressing (ELID) system. Although a nanometrically level surface was attained in this process, the system was complex and heavily relied on the operator’s expertise. Yin et al. [24,25] used ultraprecision grinding and nano-grinding techniques to fabricate WC-Co composites, and realized nanoscale surface finishes on WC-Co, but the grinding machine required a high stiffness and rigidity. Mao et al. [26] carried out chemical mechanical polishing (CMP) on a WC-12Co alloy blade and revealed the mechanism of CMP of the WC-Co alloy. You et al. [27] presented a comprehensive study of the surface-generation process of WC-0Co in laser-assisted polishing under various conditions and revealed the material-removal mechanism of the polycrystalline structure. 

Even though the polishing methods mentioned above have been successfully applied to the polishing of WC with and without the binder, the effect of the binder on the polishing process is still not clear. Moreover, our understanding of the surface integrity of WC before and after polishing requires further development. Hence, in this study, we investigated the performance of the polishing of WC with and without the binder. Due to the high hardness of WC, we decided to use a semi-rigid (SR) bonnet polishing tool [28,29] for a demonstration. Compared with the conventional pure rubber bonnet [30,31,32,33], the SR bonnet can implement higher polishing efficiency while keeping a certain degree of flexibility [34,35]. A systematic study on SR bonnet polishing of WC with and without the binder was conducted with different experiments. The effect of the binder on the material removal rate and surface and subsurface integrity are discussed.

## 2. Experiment

### 2.1. Materials

In this study, two kinds of tungsten carbide samples were used as experimental materials. Commercial high-purity binder-less tungsten carbide (hereinafter referred to as WC-0Co; produced by STC Materials LLC., purity 99.9%, theoretical density 15.64 g/cm^3^, hardness 2800 HV, average particle size about 0.6 μm) was prepared with a diameter of 40 mm and height of 10 mm. Commercial WC-12 wt.% Co (hereinafter referred to as “adhesive” or WC-12Co; brand YG12, provided by Mingyou Metal Materials Co., Ltd., (Dongguan, China) theoretical density 14.5 g/cm^3^, hardness 86~88HRA) was prepared with a size of 100 mm × 100 mm × 10 mm in a block. Before the polishing experiments, the WC-0Co was ground using an 800# diamond wheel, while the WC-12Co was roughly ground first, then lapped with 1500# diamond slurry. The surfaces of WC-0Co and WC-12Co had a 2~3 λ (λ = 632.8 nm) peak-to-valley (PV) and surface average arithmetic roughness (Sa) of 100~200 nm. Then, the samples were treated in an ultrasonic cleaner for 15 min. The cleaning process was repeated three times to thoroughly remove contaminations from the surface. Finally, the samples were dried in the air for about 10 min. The abrasive of the polishing slurry used in this study was diamond.

### 2.2. Experimental Setup

**Figure 1:**
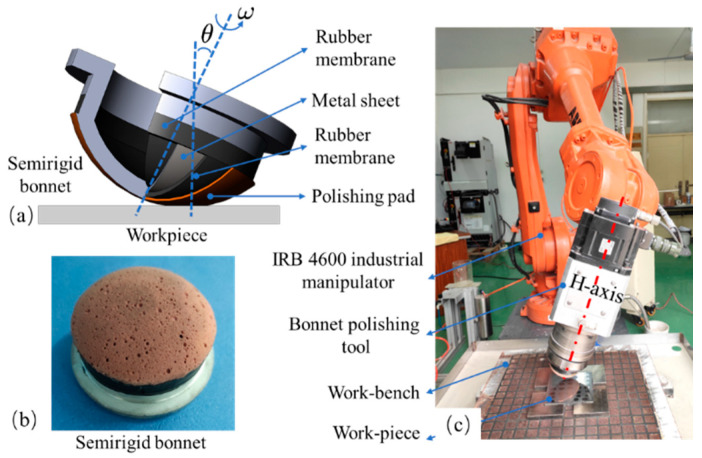
Experimental setup. (**a**) Structure of the semirigid (SR) bonnet; (**b**) photograph of the semirigid (SR) bonnet; (**c**) robot-assisted bonnet polishing system used in this study.

An SR bonnet is a spherical cap with a radius of 40 mm; the structure of the SR bonnet is shown in Figure 1a [27]. It consists of two layers of rubber membranes, embedded with a 0.3 mm stainless steel sheet, which enhances the removal efficiency and maintains certain flexibility. The outermost rubber membrane is covered with a polishing pad as shown in Figure 1b.

During polishing, the flexible rotating bonnet tool compresses the workpiece and transfers the pressure to the polishing abrasive particles between the polishing pad and the workpiece. The abrasive particles continuously scratch the surface of the workpiece, resulting in micro-/nanoscale removal of the material. Figure 1c shows the polishing system used in the experiments. Based on the principle of bonnet polishing, the polishing system is equipped with a 6-DOF ABB® robot arm (IRB4600-60/2.05) as the main body, which outfits the self-developed robot-assisted polishing device. The repeated positioning accuracy of the 6-DOF industrial robot arm is ±0.05 mm. The SR bonnet is installed at the end of the polishing setup, which is driven by a servo motor.

LP-66 (Universal Photonics, NY USA) is pasted on the SR bonnet as a polishing pad. It is a high-performance, optical-level processing material with an elastic, soft surface, pores and a long service life [36]. During polishing, the porous and soft polishing pad can effectively store the abrasive particles so the removal efficiency is high, while avoiding scratch marks on the workpiece. 

### 2.3. Experimental Design

#### 2.3.1. Comparison of Material Removal Characteristics

In the study, the tool influence functions (TIFs) of samples were obtained through two groups of experiments. Following the conditions in Table 1, the dwell times of Group 1 were 12 s, 24 s, 36 s and 48 s, while the offset was kept constant at 0.4 mm. In Group 2 the dwell time was kept constant at 36 s, and the offsets were 0.3 mm, 0.4 mm, 0.5 mm and 0.6 mm. The polishing slurry was ∼3 wt.% diamond, and the abrasive grain size was ∼5 μm. 

#### 2.3.2. Uniform Polishing Experiment

The uniform polishing experiments of WC-0Co and WC-12Co were conducted to analyze the polishing characteristics of both materials. Full-aperture polishing was carried out on the WC-0Co sample by pre-polishing followed by fine polishing. Similarly, a 15 mm × 15 mm rectangular region was polished on the WC-12Co sample by pre-polishing and then fine polishing. The raster tool path with a 1 mm scanning interval was used on both samples. The other experimental parameters are listed in Table 2. The parameters with the highest efficiency, based on the results of the TIF experiments, were used for pre-polishing, while the parameters with the best precision were used for fine polishing. After each stage of polishing, the surface roughness was measured.

### 2.4. Measurement Methods

The polished surface and subsurface morphology, surface and subsurface energy spectrum, surface chemical composition, surface roughness (Sa, Sq and Sz) and material volume removal rate (VRR) were measured and computed in this study, to evaluate the polishing performance of the SR bonnet tool on two WC materials and analyze the effect of the binder on the polishing performance. The surface of the sample was bombarded with a focused ion beam (FIB) to form a wedge-shaped groove to observe the subsurface. FIB milling (FEI Scios) was performed with a constant 30 keV, 5 nA and 0.3 nA. The subsurface morphology and energy spectrum were detected via SEM (FEI Scios). The surface morphology was measured using SEM (ZEISS sigma 500, Oberkochen, Germany). In the meantime, the surface element distribution was analyzed by an energy-dispersive X-ray spectroscope (EDS) mounted on the SEM. X-ray diffraction (XRD, Rigaku SmartLab SE, Japan) was used to analyze the surface composition of materials before and after polishing. A PGI Dimension 3 (Taylor Hobson, UK) was used for profile measurement, and a ZYGO NewView^TM^ 9000 was used to measure the 3D contours and surface roughness. 

## 3. Results and Discussion

### 3.1. Material Removal Rate Analysis

**Figure 2:**
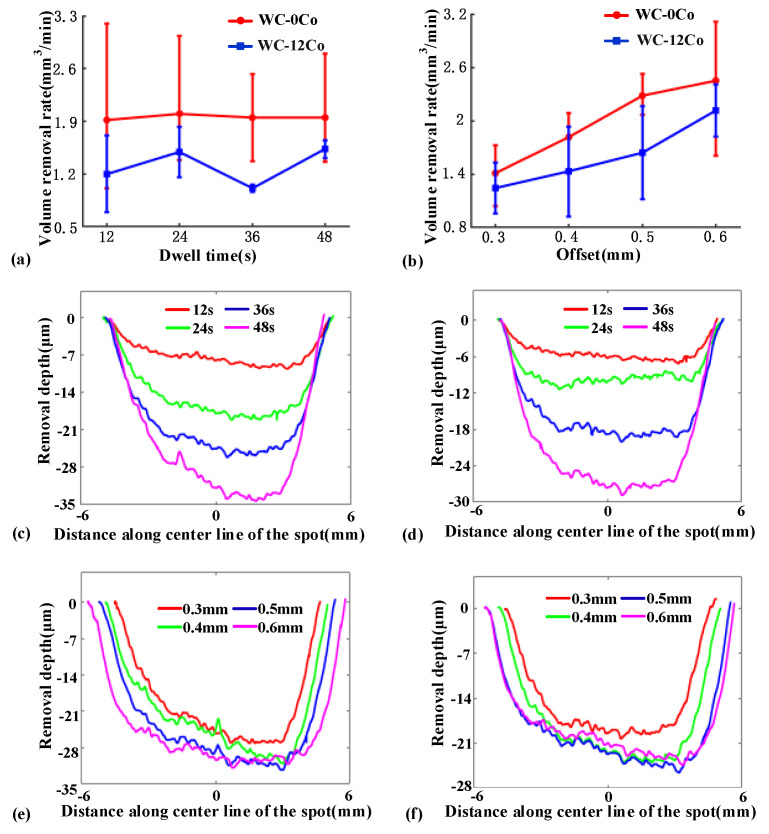
Analysis of TIFs on WC-0Co and WC-12Co under different conditions. (**a**) VRR analysis under different dwell times; (**b**) VRR analysis under different dwell times; (**c**) section profile of TIF on WC-0Co with different dwell times; (**d**) section profile of TIFs on WC-12Co with different dwell times; (**e**) section profile of TIFs on WC-0Co with different tool offsets; (**f**) section profile of TIFs on WC-12Co with different tool offsets.

The VRR variation with respect to the dwell time and offset of the two materials is demonstrated in Figure 2. It is shown that the WC-0Co has a higher VRR than the WC-12Co material under the same polishing conditions. Due to the binder, the tenacity of WC is enhanced, which possibly leads to the increase in the difficulty of material removal and the low removal rate [37,38]. It is notable that with the increase in dwell time the VRR of WC-0Co and WC-12Co materials is relatively stable, as shown in Figure 2a. Similar to previous VRR-offset investigation experiments on BK7 glass [29,39], both variation trends between VRRs and tool offsets increase gradually, as shown in Figure 2b. Figure 2c–f shows the profile shapes of polishing spots on WC-0Co and WC-12Co under different conditions. It is notable that the maximum depth of the TIF on WC-0Co is slightly larger than on WC-12Co under different conditions. According to the VRR under different dwell times, we can conclude that the average volume removal rate of WC-0Co is 1.9 mm^3^/min and that of WC-12Co is 1.3 mm^3^/min.

### 3.2. Surface Roughness Analysis inside the TIF Region

**Figure 3:**
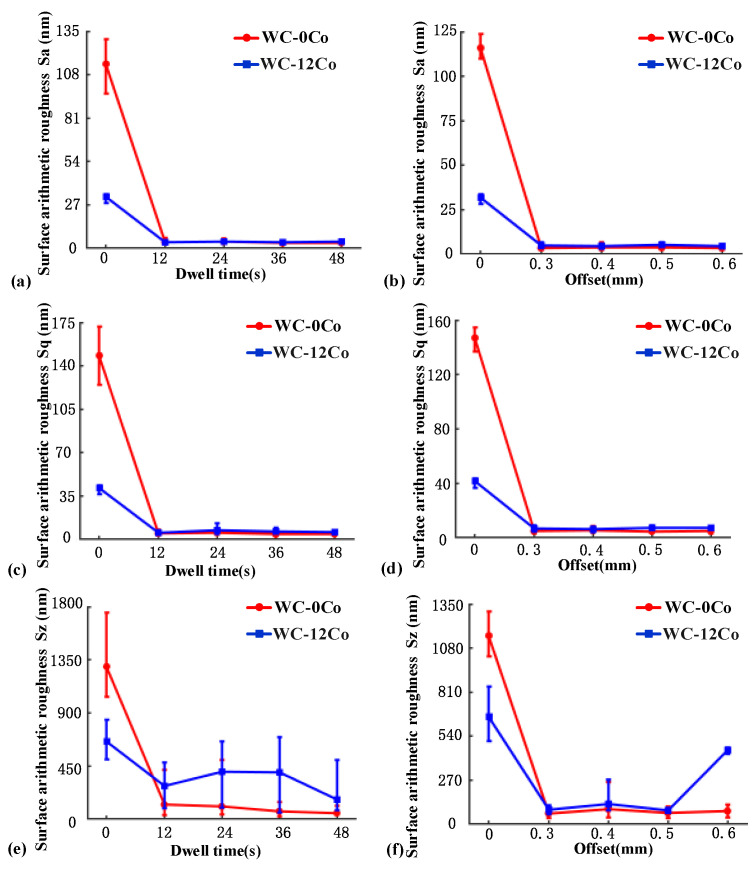
Surface roughness in Sa of the polishing spot on different materials under (**a**) different dwell times and (**b**) different offsets. Surface roughness in Sq of the polishing spot on different materials under (**c**) different dwell times and (**d**) different offsets. Surface roughness in Sz of the polishing spot on different materials under (**e**) different dwell times and (**f**) different offsets.

The relationship between the roughness (in Sa, Sq and Sz) at the center region of the polishing spots and the dwell time are shown in Figure 3a,c,e. It can be seen that the surface quality of both materials can be greatly ameliorated in a short time using the SR bonnet tool. After polishing for 12 s, the Sa of WC-0Co decreased from 114 nm to 3.7 nm and stabilized at 3.6 nm with the increase in dwell time. The Sa of WC-12Co decreased from 31 nm to 3.6 nm and stabilized at 3.9 nm. As shown in Figure 3b,d,f, the relationship between roughness and offset follows the same change trend. In addition, the surface roughness of WC-0Co is smaller than that of WC-12Co under the same parameters. The reason for this phenomenon is explained in Section 3.4.

### 3.3. Surface Roushness Analysis after Uniform Polishing

**Figure 4:**
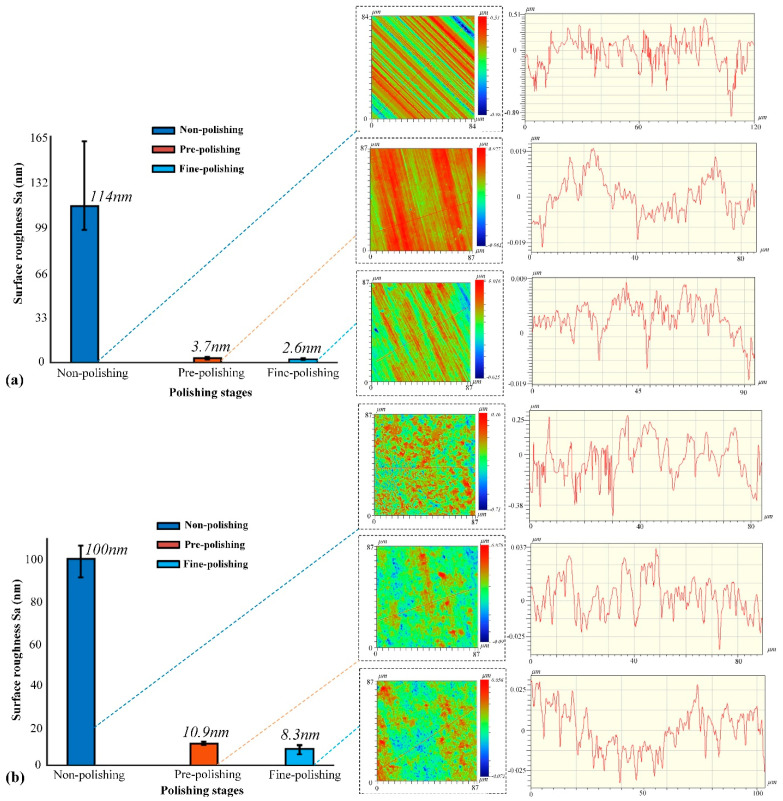
Surface roughness results at different polishing stages measured using a white light interferometer. (**a**) WC-0Co; (**b**) WC-12Co.

In addition to the TIF analysis, uniform polishing was also carried out on both WC materials to analyze the effect of the binder on the surface roughness. The surface roughness of WC-0Co polished with the SR bonnet tool is shown in Figure 4a. The results revealed that the surface roughness of WC-0Co was about 114 nm before polishing, and with different polishing stages, the surface roughness of WC-0Co decreased gradually, and converged to 2.6 nm after fine polishing. The 2D surface topography before and after every polishing stage are also demonstrated in Figure 4a. It can be observed that the surface of WC-0Co had obvious grinding tool marks and deep grooves before polishing. After pre-polishing and fine polishing, the surface scratch marks and grooves were effectively removed. It can also be seen that the surface waviness of the cross-section was reduced with different polishing stages.

The surface roughness levels of WC-12Co before and after polishing are demonstrated in Figure 4b. The surface roughness of WC-12Co was about 100 nm before polishing, while the roughness was reduced to 8.3 nm after fine polishing. From the 2D profile of the surface roughness measurement results before and after each polishing stage, shown in Figure 4b, it can be seen that the binder caused large particles to form on the polished surface, which is the main factor affecting surface roughness. At the same time, it can also be seen that the surface waviness of the cross-section was reduced with different polishing stages.

**Figure 5:**
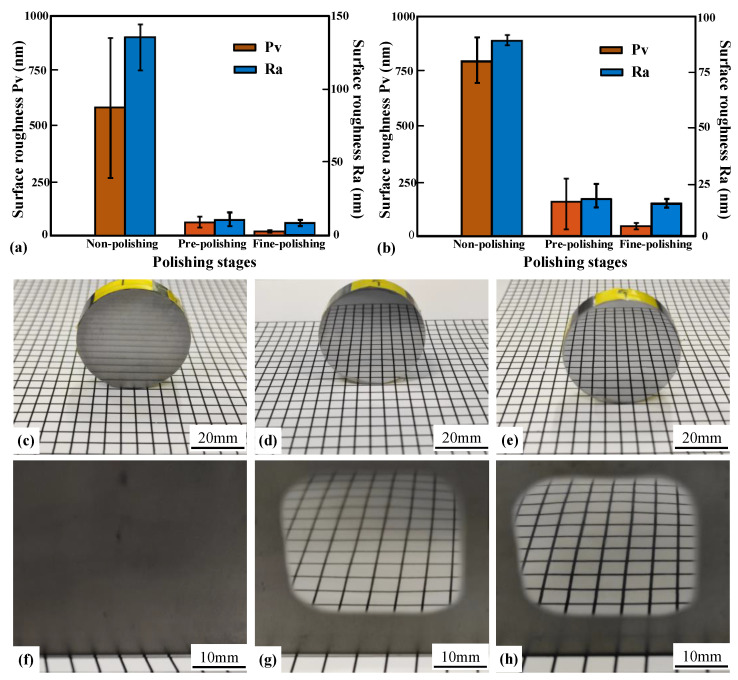
Roughness at different polishing stages measured using a PGI Dimension 3 and corresponding photographs. (**a**) Surface roughness comparison of WC-0Co at different polishing stages; (**b**) surface roughness comparison of WC-12Co at different polishing stages. Photographs of WC-0Co samples: (**c**) before polishing, (**d**) after pre-polishing and (**e**) after fine polishing. Photographs of WC-12Co samples: (**f**) before polishing, (**g**) after pre-polishing and (**h**) after fine polishing.

In addition to using a ZYGO white light interferometer to analyze the surface roughness, a Form PGI Dimension 3 was also used to analyze the surface roughness to confirm the performance. The surface roughness within 5 mm of the center area was measured, and the results can be found in Figure 5. It can be seen that the central roughness levels in arithmetic mean roughness (Ra) and peak-to-valley (Pv) values of the two materials were effectively reduced through different polishing stages. Photographs of WC-0Co and WC-12Co surfaces before and after polishing are shown in Figure 5c–h. Both materials have been successfully polished to obtain a super smooth, mirror-like surface.

### 3.4. Surface and Subsurface Topography Analysis of Tungsten Carbide before and after Polishing

**Figure 6:**
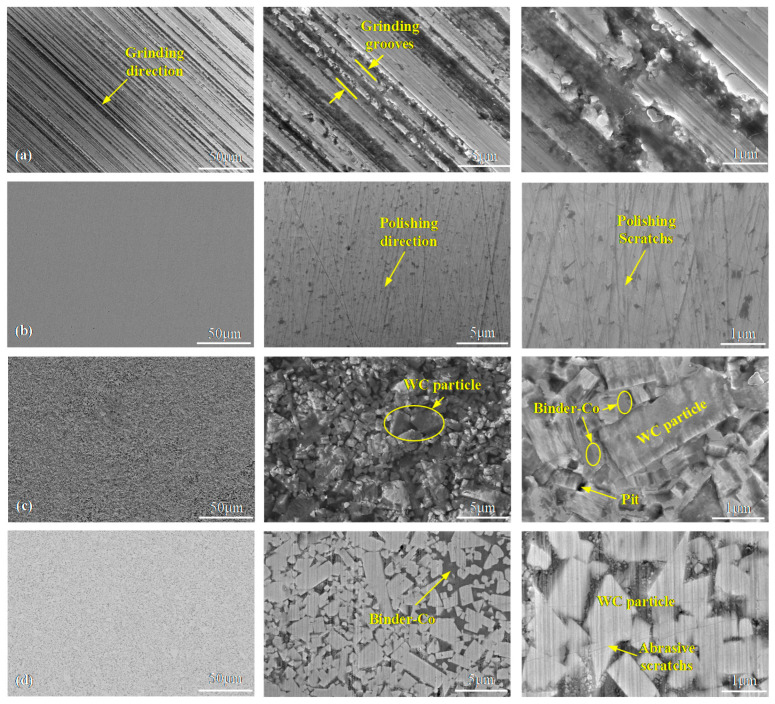
SEM images of WC-0Co and WC-12Co before polishing and after fine polishing in three different magnifications. (**a**) WC-0Co before polishing; (**b**) WC-0Co after fine polishing; (**c**) WC-12Co before polishing; (**d**) WC-12Co after fine polishing.

SEM images with different magnifications of WC-0Co and WC-12Co before polishing are shown in Figure 6a,c. Obvious grinding marks can be observed on WC-0Co before polishing, which was conducted using an 800# diamond grinding wheel. The surface of WC-12Co treated by grinding and lapping has no obvious machining marks. However, clear and irregular WC particles can be seen on the surface of WC-12Co before polishing. The size of the WC particles varies from smaller than 1 μm to ~6 μm. Most of them are prismatic structures. SEM surface images of WC-0Co and WC-12Co after fine polishing are shown in Figure 6b,d. It can be seen that WC-0Co obtained a highly smooth surface after polishing. The grinding marks were thoroughly removed, replaced by much shallower and smaller abrasion marks generated by the polishing process. Similarly, the surface of WC-12Co was also smoothed, leaving abrasion marks on the WC particles. However, obvious pits and unevenness can be found between WC particles and binders, which is the reason why the surface roughness of WC-12Co after fine polishing is greater than that of WC-0Co.

**Figure 7:**
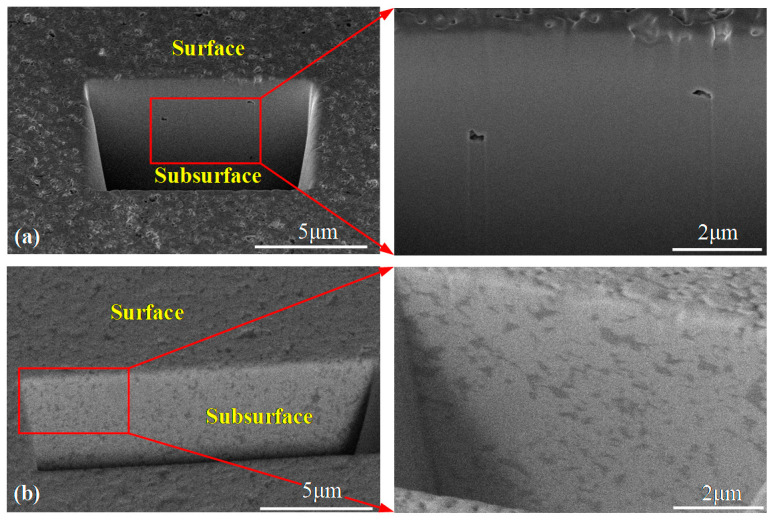
Subsurface SEM images of WC-0Co and WC-12Co after polishing, prepared with an FIB. (**a**) WC-0Co; (**b**) WC-12Co.

In order to analyze and compare their subsurface damage after polishing with the SR bonnet tool, cross-sectional surfaces of the samples were prepared using an FIB, as shown in Figure 7. The results revealed no obvious defects or cracks in the subsurface area of WC-0Co or WC-12Co after polishing with the SR bonnet tool. In addition, it was also observed that WC-0Co had a much better surface quality both on the surface and subsurface than WC-12Co on the whole.

### 3.5. Surface Material Composition Analysis before and after Polishing

**Figure 8:**
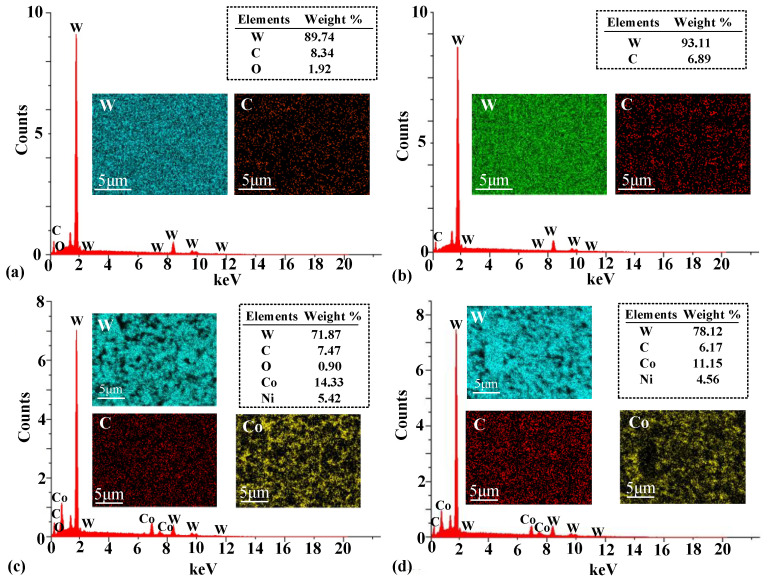
EDS analysis results. (**a**) WC-0Co before polishing; (**b**) WC-0Co after fine polishing; (**c**) WC-12Co before polishing; (**d**) WC-12Co after fine polishing.

In order to investigate a possible chemical reaction during SR bonnet polishing of WC-0Co and WC-12Co, samples before and after polishing were analyzed using EDS. Figure 8 shows the EDS results for the WC material. WC-0Co before polishing was revealed to have an oxide layer produced from the grinding process since elemental oxide was detected, as shown in Figure 8a. The oxide layer was successfully removed after SR bonnet polishing, visible as the lack of elemental oxide shown in Figure 8b. The same phenomenon was also observed on the WC-12Co surface before and after polishing. Elemental Ni was also detected in WC-12Co, induced by the impurity of the sintering process. 

**Figure 9:**
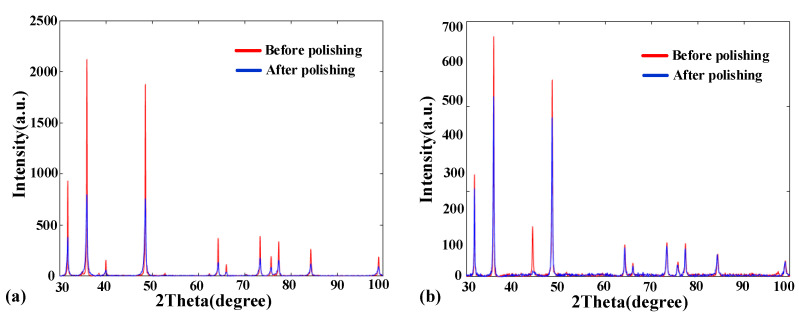
XRD results of typical areas of surface with (**a**) WC-0Co; (**b**) WC-12Co.

To determine the possibility of a material phase change during SR bonnet polishing, the contact pressure during which is much larger than traditional rubber bonnet polishing, an XRD analysis was carried out on the surface before polishing and after fine polishing of WC-0Co and WC-12Co. As shown in Figure 9, the peak positions of the XRD curves of WC-0Co and WC-12Co after fine polishing are almost the same as their XRD curves before polishing. Hence, no phase change occurred during polishing of the materials using the SR bonnet tool. 

### 3.6. Discussion

**Binder affects material removal rate.** The material removal rate of WC-12Co is lower than that of WC-0Co when polishing using diamond slurry, induced by the high viscosity of the binder material Co. The polishing abrasive used in this study was diamond, the hardness of which is much higher than both WC and Co. However, the viscosity of the material Co can reduce the material removal rate during polishing, which was proven by the results of VRR comparison between WC-0Co and WC-12Co, as shown in Figure 2. Nevertheless, if the hardness of the polishing abrasive is lesser than WC but greater than Co, the material removal of WC will become difficult. Since WC is the main component, the material removal rate of WC will directly determine the material removal rate with the Co binder. Hence, the material removal rates when polishing WC with different percentages of binder are different. In addition, we must note that different grain sizes of WC were used in WC-0Co and WC-12Co, which may have affected the above conclusion. The grain size of WC in WC-0Co was 0.6 μm, while it was 1~6 μm in WC-12Co. According to reference [40], a smaller grain size of WC corresponds to greater hardness of the WC-Co material. This means that the hardness of WC-12Co using the grain size of 0.6 μm was greater compared to the material using the grain size of 1~6 μm. Nevertheless, the material removal rate when polishing WC-12Co was still less than WC-0Co, which further proves that the binder Co can reduce the material removal rate during SR polishing using diamond slurry to a certain degree.

**Binder affects surface quality**. As shown in Figure 7, WC-0Co has much better surface and subsurface qualities than WC-12Co because of the extremely dense microstructure of the sintered WC. In this study, the best roughness of Sa 2.6 nm was obtained on WC-0Co, while it was Sa 8.3 nm on WC-12Co. For WC-0Co, the material removal rate can be maintained evenly across the whole surface to obtain an ultra-smooth surface. Meanwhile, the material removal rate on WC-12Co is different at different positions, induced by the different materials WC and Co. Hence, pits and unevenness can be commonly observed on the polished WC-12Co surface, as shown in Figure 6, leading to worse surface roughness. Since 5 μm diamond abrasive was used in this study for polishing, we propose that the surface roughness could be further improved by using finer or nanometer-scale polishing abrasives.

## 4. Conclusions

In this paper, the effect of the binder during polishing of tungsten carbide (WC) material was demonstrated, as well as the polishing performance of a semi-rigid (SR) bonnet tool, on two WC materials with and without the binder. Characterization and comparison of the material removal as well as the surface integrity before and after polishing were conducted on binder-less tungsten carbide (WC-0Co) and tungsten carbide with 12 wt.% of cobalt (WC-12Co) through a series of experiments on a 6-DOF robotic polishing device. The material removal rate and surface roughness before and after polishing were compared through experiments of TIFs, and the polished surface integrity was inspected through uniform polishing experiments. The following conclusions can be drawn.

1. Both WC-0Co and WC-12Co materials can been successfully polished to obtain nanometric surface roughness using an SR bonnet tool;
2. The material removal rate of WC-0Co is higher than that of WC-12Co, as we concluded after comparing the volume removal rates of their TIFs when using diamond polishing slurry, with removal mainly induced by the effect of the binder Co;
3. Better surface roughness can be obtained on WC-0Co than WC-12Co, indicating that the introduction of the binder is not beneficial to obtain extremely smooth surfaces;
4. No obvious chemical reaction or phase change of the surface were observed during polishing of WC-0Co and WC-12Co using the SR bonnet tool;
5. No obvious damages to the subsurface layer was observed after polishing WC-0Co and WC-12Co using the SR bonnet tool according to SEM photographs. Similarly, no element changes were observed in the subsurface layer according to the EDS analysis.

## Figures and Tables

**Table 1:** TIF generation conditions.

	Group 1				Group 2			
	1	2	3	4	1	2	3	4
Dwell time (s)	12	24	36	48	36			
Tool offset (mm)	0.4				0.3	0.4	0.5	0.6
Others	$\theta =20^{{}^{\circ}}$, inner pressure = 0.2 MPa, ω = 500 rpm							
Experimental materials	WC-0Co and WC-12Co							

**Table 2:** Uniform removal conditions.

Conditions	Pre-Polishing	Fine Polishing
Tool radius (mm)	40	40
Precession angle (deg)	25	10
Inner pressure (Mpa)	0.25	0.1
Speed (rpm)	1000	500
Offset (mm)	0.6	0.4
Feed rate (mm/min)	100	100
Polishing slurry	~3 wt.% diamond, 5 μm on average	
Experimental materials	WC-0Co and WC-12Co	

## Data Availability

Not applicable.
